# Cultural practices, wildlife trade dynamics, and climate-migration interactions: an integrated one health approach to reducing zoonotic spillover risks in Africa

**DOI:** 10.3389/fvets.2026.1900536

**Published:** 2026-09-07

**Authors:** Benjamin Obukowho Emikpe, Jude Dzevela Kong, Dickson Shey Nsagha, Sodiya Adesina, Gelan Ayana, David Niyukuri, Elia Badjo, Raphael Deladem Folitse, Abdul Rahman Yakubu, Prince Nana Takyi, Sampson Yeboah, Sunday Ochonu Ochai, Ziekah Yiryele Meyir, Kafui Kwasi Dzasi

**Affiliations:** 1 Department of Pathobiology, School of Veterinary Medicine, Kwame Nkrumah University of Science and Technology (KNUST), Kumasi, Ghana; 2 Africa-Canada Artificial Intelligence and Data Innovation Consortium (ACADIC), Ontario, ON, Canada; 3 Global South Artificial Intelligence for Pandemic and Epidemic Preparedness and Response Network (AI4PEP), Toronto, ON, Canada; 4 Department of Epidemiology and Biostatistics, School of Public Health, Kwame Nkrumah University of Science and Technology (KNUST), Kumasi, Ghana; 5 Department of Health Policy, Management and Economics, Kwame Nkrumah University of Science and Technology (KNUST), Kumasi, Ghana; 6 International Centre for Antimicrobial Resistance Solutions (ICARS), Copenhagen, Denmark; 7 Department of Veterinary Tropical Diseases, Faculty of Veterinary Science, University of Pretoria, Pretoria, South Africa; 8 Zoological Gardens, Wildlife Division, Forestry Commission, Kumasi, Ghana; 9 Global Health Security, Accra, Ghana

**Keywords:** climate change, human migration, one health, wildlife trade, zoonotic spillover

## Abstract

The drivers causing spillovers of zoonotic diseases in Africa are multifaceted and include cultural and traditional wildlife practices, illegal trade channels, environmental factors driven by climate change, and human migration. These drivers have been assessed separately in various reviews, and no synthesis has integrated all four within a unified multistage zoonotic spillover framework in Southern Africa, West, and Central Africa. This narrative review explores how cultural practices, the trading of wildlife, climatic changes, and forced human migration contribute to the zoonotic spillover risks. The literature search was conducted with PubMed/MEDLINE, Scopus, Web of Science, and Google Scholar databases between the years 1990 and 2026 using a structured search strategy. Bushmeat consumption, zootherapeutic practices, and wildlife trade create direct and sustained wildlife-human contact pathways, with zootherapeutic handling chains being the most poorly monitored exposure pathway. Climate-induced range shifts and habitat loss are increasing reservoir host distribution into human-adjacent environments, and climate-induced migration is moving reservoir populations to new areas without known exposure norms. Collectively, this evidence suggests that these drivers do not work in isolation at their intersections, creating compounding risks that cannot be solved by a single-domain intervention. We present this as an interpretive synthesis of the evidence reviewed, not a directly tested findings. Three documented areas highlight the need for strengthening the current implementation of One Health: enforcement measures that push trade into clandestine channels, underfunded wildlife surveillance, and the lack of community knowledge systems in the approach to designing surveillance. Women in urban bushmeat retail, a structurally high-exposure population, remain absent from formal serological surveillance programs. On the basis of these documented gaps, the review recommends redesigning surveillance for the prevention of zoonotic spillover, including sentinel systems in Sahelian and forest-margin areas linked to climate. Structural One Health governance reform will also be needed to implement these priorities, namely, incorporating community knowledge systems, climate, and migration data into national surveillance systems.

## 1 Introduction

Spillover of zoonotic diseases remains one of the most important public health issues on the African continent due to complex interactions between humans, wildlife, and the ongoing changing of ecosystems (1, 2). Zoonotic spillover is the spread of pathogens from non-human animals to humans, typically through increased contact at the human-animal-environment interface (3). Zoonotic spillover is a process that is not random but composed of four successive conditions: pathogen occurrence in a reservoir host, opportunity of contact between the reservoir host and human, vulnerability of humans to infection, and the ability of onward transmission among the human population (4–6). Throughout this review, this multistage framework is used as an organizing principle, with each section examining how cultural practices, wildlife trade, climate change, and human migration interact with one or more of these conditions as a way to increase the risk for zoonotic spillover.

Ecological disruption, biodiversity loss, wildlife use, and increased human activity are factors that increase the contact rate and likelihood of zoonotic spillover (7, 8). Wildlife hunting, handling, eating, trading, and transporting wildlife products are direct exposures to zoonotic viruses. Climate change may also increase the risk of zoonotic spillovers due to a shift in species distribution, movement corridors for wildlife, and changes in pathogen ecology (9–11).

There have been several zoonotic spillover events in Africa that demonstrate the complexity of human–animal–environment interactions. The 2013–2016 Ebola Virus Disease (EVD) outbreak in West Africa, which began in Guinea and spread to Sierra Leone and Liberia, was linked to human exposure to wildlife, primarily bats in forest fringe communities (12, 13). Lassa fever outbreaks in Nigeria may be linked to the increased interaction between humans and rodent reservoirs (*Mastomys natalensis*), and are associated with poor housing conditions, poor food storage practices, and seasonal changes in the ecology (14). Hunting and the consumption of bushmeat in forests have been associated with multiple outbreaks of Ebola in Guinea and the Democratic Republic of Congo (15, 16). Climate-related flood events have been linked to Rift Valley fever outbreaks in Kenya, as they increase the mosquito population and increase the risk of transmission from animals to humans (17, 18). The examples show that zoonotic spillovers are specific to the local context, but have a recurring theme: environmental change, animal movements, cultural practices, and poor surveillance systems on the continent.

Recent zoonotic outbreaks show sustained human-to-human transmission can occur after an initial event. The 2026 Bundibugyo virus disease outbreak in the Democratic Republic of the Congo, which has spread to Uganda, illustrates the potential for zoonotic pathogens to cause international human transmission (19). The mpox outbreak in Africa that occurred in multiple countries is also an example of a zoonotic pathogen that can initiate transmission in human populations and sustain human-to-human transmission across networks of close physical contact and sexual activity (20, 21).

Existing reviews have provided valuable insights into specific factors contributing to the spread of zoonotic pathogens in Africa. Several have focused on the specific pathogens and their ecological determinants (22, 23), others have explored the importance of bushmeat hunting and wildlife trade in pathogen transmission (24, 25), the contribution of climate variability to the dynamics of vectors and wildlife-associated disease (26, 27), and the use of One Health frameworks to zoonotic disease surveillance (28, 29). Although several prior analyses have considered several drivers, none have reviewed cultural practices, wildlife trade, climate-induced ecological alterations, and human migration in the same multistage zoonotic spillover framework for West, Central, and Southern Africa. This review, for the first time, investigates the interlinked social, ecological, and demographic components throughout the phases of zoonotic spillover and provides the framework to develop One Health intervention options that address the combined components, rather than the individual components. Thus, with this approach, the review should be able to identify intervention options that would otherwise go unrecognized.

The different domains have varying quality and quantity of supporting evidence. Greater evidence and evidence synthesis exist concerning the relationship between bushmeat consumption and trade, and zoonotic spillover and exposure. Less evidence exists concerning climate-induced migration pathways, the relationship of migration to spillover, and governance structures to mitigate spillover risk. In these domains, evidence supports emerging, indirect, observational, or conceptual evidence. This review aims to describe the level of empirical evidence that supports the remaining plausible conclusions concerning the relationship of the domains and their zoonotic spillover, and other domains that lack sufficient evidence to support direct conclusions.

**Figure 1:**
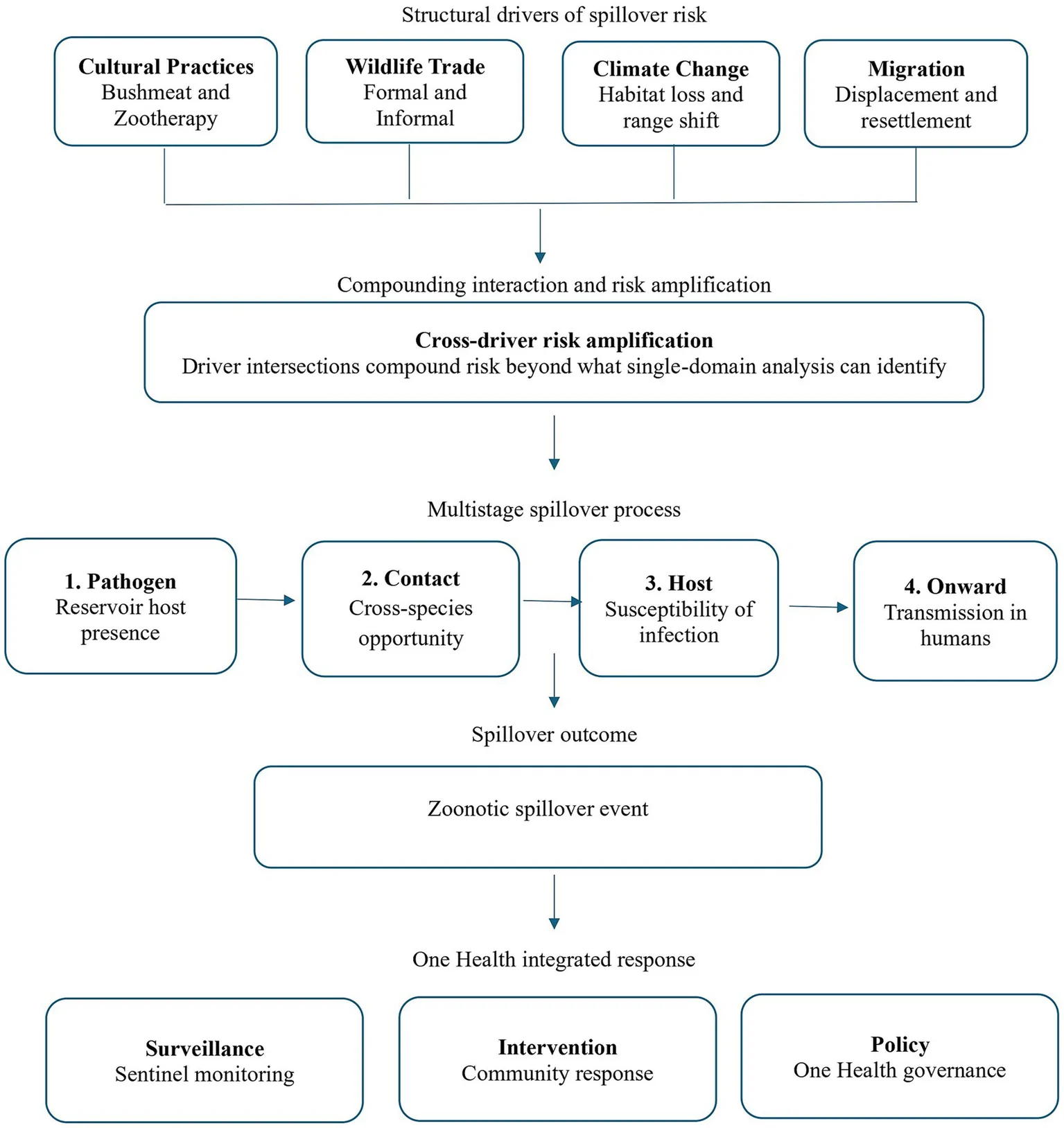
One health–based conceptual framework of drivers and pathways influencing zoonotic spillover in Africa.

This review aims to address that gap, with six specific objectives: (1) to review the influence of cultural practices, such as bushmeat consumption, traditional medicine practices involving wildlife, and ritual handling of animals, on reservoir contact rates and exposure from reservoir hosts across African subregions; (2) to analyse how formal and informal wildlife trade networks create and expand contact opportunities between reservoirs and humans, and how they facilitate cross-species transmission of pathogens; (3) to review the influence of climate-driven ecological changes, including changes in host distribution from reservoirs and vector dynamics driven by habitat loss, species range shifts, and extreme weather events on zoonotic spillover; (4) to examine how migration and displacement interact with these ecological and trade-related drivers to extend the geographic reach of zoonotic spillover risk; (5) to critically assess current One Health implementation efforts in Africa, identify documented areas in need of strengthening, and propose evidence-based and context-differentiated interventions to mitigate zoonotic spillover risk at the intersections of these drivers; and (6) to examine how these drivers interact and may reinforce one another, creating compound spillover pathways that may not be fully captured by analyses focused on individual risk domains (Figure 1). This review seeks to highlight potential integrated intervention points from a One Health perspective that have been missed by analyses in any single domain, by demonstrating how these objectives are interconnected and driven by the multistage process of zoonotic spillover, with a specific focus on structural social, economic, and environmental determinants.

## 2 Methods

### 2.1 Study design

This review uses a broad integrative narrative approach to synthesise published evidence on the cultural, ecological, trade, and climatic determinants of zoonotic spillover risks in Africa. Narrative reviews are suitable for interdisciplinary questions covering multiple disciplines, evidence types, and geographical settings, when the aim is not to numerically add up a homogeneous set of studies, but to synthesize and conceptualise the evidence (30, 31). This method was chosen to ensure that the review covered cultural anthropology, disease ecology, wildlife trade governance, and climate science, aspects that are not possible to cover in a systematic review or meta-analysis due to strict eligibility criteria. This review integrates evidence from 124 sources. These include peer-reviewed articles, primary studies, systematic and narrative reviews, and reputable institutional reports. Identified through structured searches with citation chaining, synopses of systematic searches are provided for readers.

To have more transparency and reproducibility, a structured approach was used to identify, select, and synthesise relevant literature. The databases, search terms, publication period, eligibility criteria, screening procedures, and citation-tracking methods were clearly defined. Although this was not a systematic review, studies were selected according to the review objectives, thematic relevance, and African subregional context to minimise arbitrary selection.

### 2.2 Search strategy

Four established academic databases (PubMed/MEDLINE, Scopus, Google Scholar, and Web of Science) were used for a structured search of the peer-reviewed and grey literature. The choice of these databases was based on their coverage of biomedical, ecological, social science, and interdisciplinary literature. In the first stage of searching, no language restrictions were placed. When determining the final inclusion of non-English documents, the reviewers’ (BOE, JDK, DSN, SA, GA, DN, EB, RDF, SOO, ZYM) ability to evaluate documents in English was taken into account. The inclusion of non-English documents was based on the availability of English translations or the presence of documents in English that would support the evaluation of the relevance and primary conclusions of the non-English documents.

**Figure 2:**
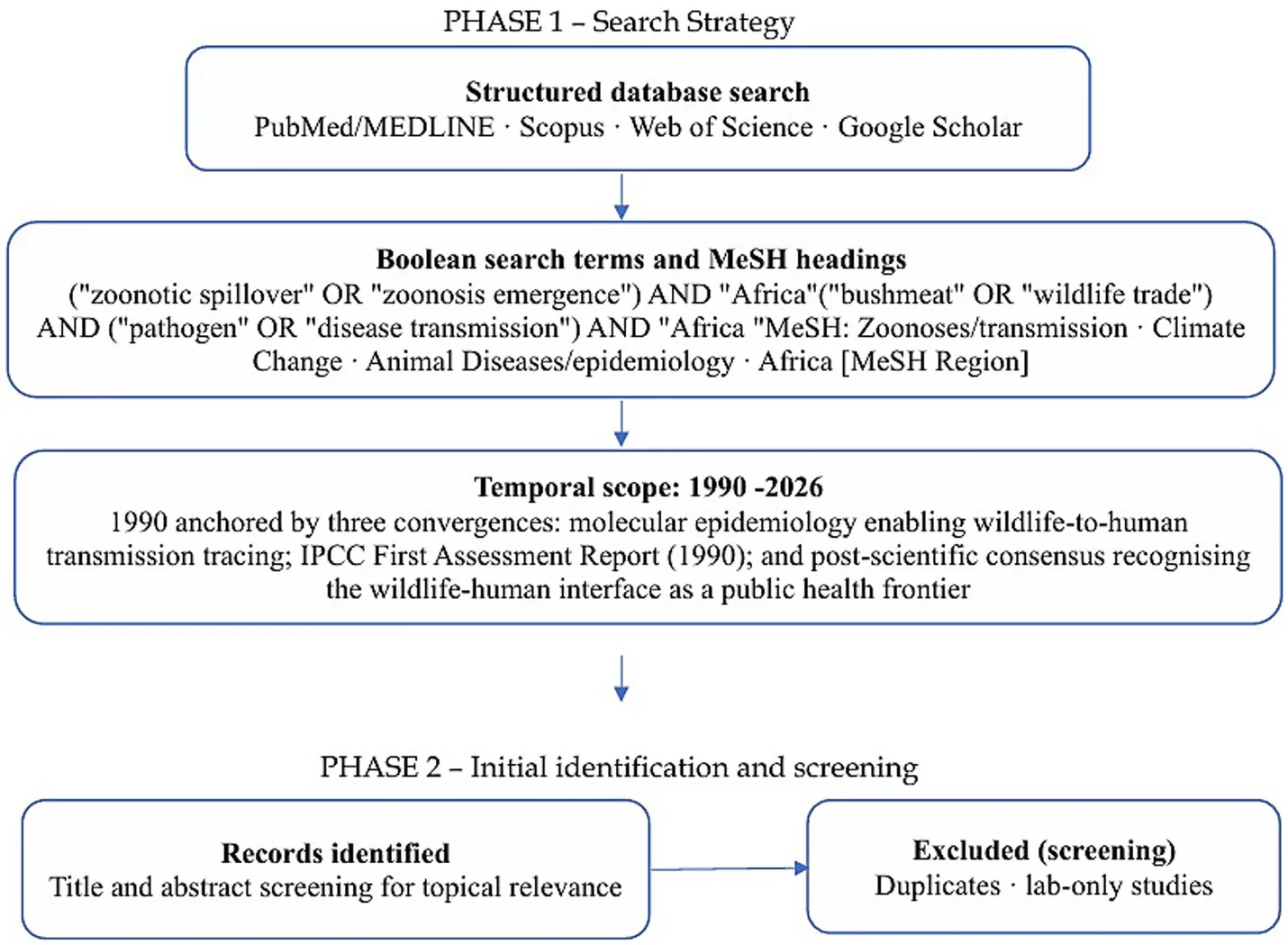
Flow diagram of the literature search, initial identification, and screening process.

The searches were done in an iterative way, between June and July 2026, and the last search was done on 20th July 2026. The period of time covered was 1990 to 2026. This starting point reflects the three developments which characterized the modern era of zoonotic spillover risk: (i) the emergence of molecular epidemiology as a tool for tracing wildlife-to-human transmission; (ii) the publication of the IPCC First Assessment Report (32), which established the scientific consensus on anthropogenic climate change that now shapes contemporary climate-disease linkage research, and (iii) the adoption of Convention on Biological Diversity (33) and the wider international recognition of the structural role of biodiversity loss in driving emerging infectious diseases. Evidence preceding 1990 is unlikely to reflect the ecological, epidemiological, and institutional conditions under which contemporary zoonotic spillover risk in Africa operates (Figure 2).

Search terms were combined using Boolean operators (AND, OR) across title, abstract, and keyword fields. Key terms in the search strings: (“zoonotic spillover” or “emergence of zoonosis”) AND “Africa”; (“bushmeat” or “wildlife trade”) AND (“pathogen” or “disease transmission”) AND “Africa”; (“zootherapy” or “traditional medicine”) AND (“zoonotic” or “wildlife”) AND “Africa”; (“climate change” or “habitat loss” or “range shift”) AND (“zoonotic disease” or “spillover”) AND “Africa”; (“human migration” or “displacement”) AND “zoonotic” AND “Africa”; and “One Health” AND (“spillover” or “wildlife”) AND “Africa.” When available, Medical Subject Headings (MeSH) terms were used in the PubMed searches, such as: Zoonoses/transmission, Animal Diseases/epidemiology, Climate Change, Conservation of Natural Resources, and Africa [MeSH Region]. Database searches were supplemented by citation chaining to find other relevant sources.

Sources were limited to those published between 1990 and 2026. No restrictions were made regarding the relevance, methods or institutions of sources. No restrictions were made regarding study design, the sub-region of Africa where the studies were conducted, or the use of qualitative or quantitative methods. The aim of the review was to collect and synthesize the evidence from diverse disciplines and different methodological approaches.

Search results were scanned for duplicates. If a study was found multiple times, in database searches or citation searches, all records were removed except one. Citation searches were performed in two ways. The first was a backward citation search, in which evidence cited by a study or review was located. The second was a forward citation search, in which the study was located if it was cited by the key study. Eligibility of cited evidence was assessed, and the evidence was subjected to the database search.

### 2.3 Source inclusion

Due to the focus of this review on the breadth of synthesis rather than a fixed, protocol-defined corpus, the decision to include items was based on relevance and analytical contribution. The integrative, multi-thematic approach of the review resulted in the selection of sources that covered one or more of the following topic areas: zoonotic spillover or wildlife-associated pathogen transmission; bushmeat consumption, hunting, or wildlife trade; zootherapeutic practices and public health implications; climate-driven ecological change and its implications for disease dynamics; human migration, displacement, or mobility in the context of zoonotic risk; or One Health frameworks and their application in African or directly comparable settings.

The preliminary screening included multiple phases. In the initial phase, duplicate records were eliminated. At the second phase, researchers scrutinized titles and abstracts of records for their pertinence to the review’s thematic domains. Records for which relevance was inconclusive were retained for further evaluation. In the final phase, potentially eligible sources underwent full-text evaluation for the review’s inclusion and exclusion criteria. Therefore, pertinent sources went beyond the existence of a relevant keyword or topic. Sources were evaluated for their relevance to the review’s conceptual framework, human-animal-environment interactions, the methods and approaches to evidence, the findings’ frameworks and institutions, and the findings themselves.

**Figure 3:**
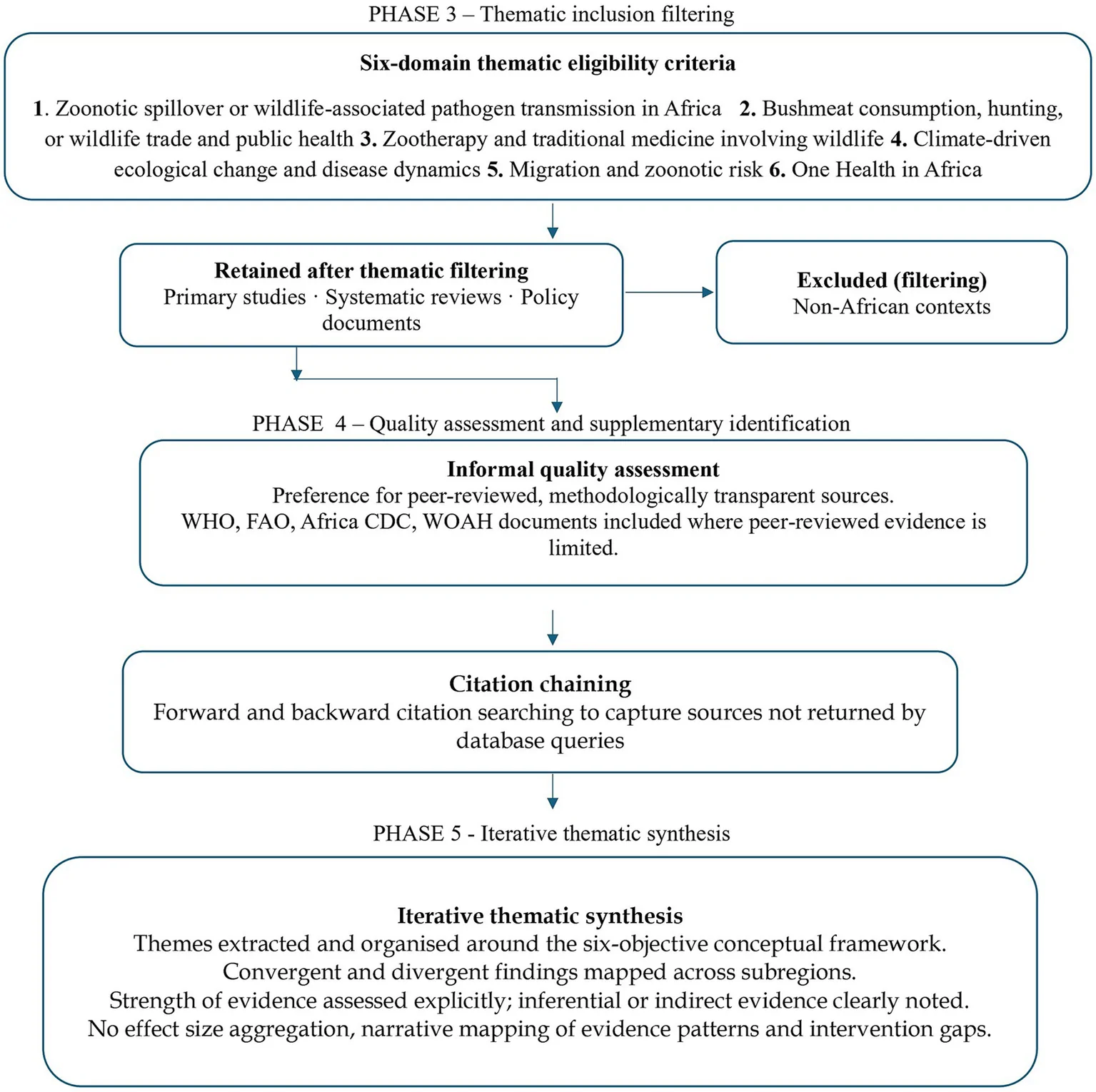
Flow diagram of the thematic inclusion and quality assessment.

Both empirical primary studies (quantitative and qualitative) and authoritative secondary sources, including systematic reviews, technical reports, and policy documents from internationally recognized institutions (WHO, FAO, Africa CDC, WOAH), were eligible for inclusion. Purely laboratory-based studies examining pathogen biology in isolation, without reference to human exposure pathways or ecological context, were excluded, as were studies focused entirely on contexts outside Africa with no direct analytical relevance to the review’s framework (Figure 3). The final synthesis balances evidence’s strengths and weaknesses and does not assume all sources have equal evidentiary weight.

Evidence included here was not assigned a single, definitive quantitative quality score. Evidence was considered for its nature and strength. Findings supported by multiple independent empirical studies and systematic reviews, alongside robust epidemiological evidence, were seen as more informative than findings supported by individual empirical studies, institutional reports, grey literature, and conceptual frameworks. Institutional reports were mainly cited to provide the most up-to-date epidemiological data and data from surveillance and reports. Grey literature and conceptual sources were cited to provide framing for newly identified issues and to articulate research gaps. The strength of the conclusions was evaluated based on the quantity, consistency, and quality of the available evidence.

The lead reviewers (BOE, JDK, DSN) completed the screening process and made calls on borderline and major source conclusions in the presence of other reviewers (SA, GA, DN, EB, RDF, SOO, ZYM). If conflict on eligibility or interpretation arose, the source was examined again, and a consensus was achieved through discussion. This was done to alleviate the concern that relevance was judged and inclusion calls were made through the lens of one reviewer.

Different methods were employed to assess for selection bias. The first involved a comprehensive search that included multiple databases. The second involved using different search terms to cover gaps for descriptors across other disciplines. The third was conducting backwards and forward citation searches and reviewing literature that may have been omitted in the primary search. The fourth was consideration of both qualitative and quantitative studies. The final step was in line with the first, considering the main thematic areas in the conceptual framework. This was done rather than relying on studies which the authors preferred.

### 2.4 Quality assessment and data synthesis

The quality of the sources was evaluated informally and deliberately, with preference given to peer-reviewed articles, qualitative research with explicit methodology, and documents from internationally recognized health organizations. Grey literature and non-peer-reviewed sources were included only where they provided unique programmatic, regional, or contextual evidence unavailable in the peer-reviewed literature, and this is noted where relevant. The quality and origin of the evidence were important in assessing the findings. Conclusions based on many independent empirical studies, systematic reviews, and strong epidemiological evidence were considered more sound than conclusions based on individual studies, institutional reports, the grey literature, and/or conceptual frameworks. Where evidence was indirect, inferential, or biologically plausible, and/or primarily based on expert or conceptual sources, the conclusions presented more cautiously.

Because the multi-disciplinary scope of this review required drawing on evidence spanning cultural anthropology, disease ecology, trade governance, climate science, and migration studies, no formal exclusion threshold based on study design was imposed. Instead, the strength and quality of evidence presented for each analytical claim is made explicit across the review, and when the evidence is indirect or inferential, or only biologically plausible evidence for an association is presented, this is made clear. Thematic data extraction was done based on the six-objective conceptual framework of the Review. Synthesis was conducted iteratively, with themes refined as additional literature was identified through citation searching. This review does not make quantitative estimates or attempts to pool effect sizes, but rather describes patterns of evidence, identifies areas of convergence and divergence within subregions, and emphasizes gaps in surveillance and interventions with practical implications for public health policy and One Health programming (Figure 3).

## 3 The historical and cultural bases of bushmeat consumption

The consumption of bushmeat in Africa is deeply embedded within cultural, nutritional, and socio-historical contexts, and a meaningful step toward reducing the zoonotic risk of spillover will start by gaining a thorough understanding of the underlying determinants. Historically, regions with scarce livestock density have relied on wild game as a source of animal protein (34, 35), and, over time, game hunting and consumption evolved to form part of social ceremonies, passage rites, and status displays. While these practices continue to exist today, the modern-day persistence of these practices reflects a complex interplay of cultural, economic, and ecological factors rather than a simple continuation of traditional practices.

This literature shows that there is a significant tension that remains under-resolved. Many studies conducted in West Africa, including those by van Vliet *et al*. (36) and Chausson *et al*. (37), document grasscutters, giant pouched rats, antelopes, and bats as socially valued foods consumed at parties, ceremonies, and urban restaurants across Côte d’Ivoire, Sierra Leone, Nigeria, and Ghana. Within this body of literature, bushmeat is often described as an identity, culinary preference, and cultural belonging. However, results from Ordaz-Nemeth *et al*. (38) on behavioral responses for the West African Ebola outbreak indicate a more complex interpretation. Their study showed that cultural attachment was not the only factor that influenced whether people decreased their consumption of bushmeat during a time of increased health risk; household wealth and education were significant predictors. These findings indicated that, in some urban contexts, beyond cultural embedding, economic positioning, and consumer choice, certain factors can also have an impact on patterns of consumption. Consequently, an intervention focusing mainly on cultural sensitivity might not consider the relevance of the economic substitution as a potentially more effective means for behavior change.

In Central Africa, the evidence supporting deeper cultural embeddedness seems to be substantial. Forest-dependent communities in Cameroon, Gabon, and DRC rely heavily on non-human primates, small forest antelopes (duikers), and pangolins for livelihood, and these practices are embedded in complex social and ecological systems, and cannot readily be replaced by market alternatives (35, 39). In such an environment, the risk of a zoonotic spillover may stem from deeper issues, as hunting, slaughtering, butchering, and the consumption of these animals entail repeated exposure to blood, body fluids, and tissues of the animals (40–42). However, in Southern Africa, the dynamics are quite different. Consuming wildlife meat is not widespread; the practice is often illegal and more associated with masculine identity than with daily subsistence, which means that the problem relates more to clandestine trade than cultural norms (43, 44).

Regional differences have a practical significance that has been overlooked in literature. It is important to understand that studies that view the consumption of bush meat as single Africa-wide cultural phenomenon may fail to capture the significant differences that may exists, and consequently, the proper response needed. Where economic factors are the main factor, an intervention that increases access to low-cost protein sources and diversifies livelihoods might be most relevant. Community co-design and participation with social structures and traditional leaders will likely be needed where bushmeat use is deeply rooted in cultural and traditional practices. Where consumption is mostly obtained through clandestine trade networks, enforcement efforts can be needed but must be complemented by trade network analysis and provision of alternative livelihoods, to prevent displacement of trade into less monitored channels. Importantly, evidence already exists that broad, culturally insensitive bans often lead to resistance, to a shift of trade to less visible channels, and to unintended public health consequences (37, 45, 46). A lesser addressed question is how much these interventions might be unsuccessful due to a lack of alignment with the underlying factors driving bushmeat consumption.

## 4 Socioeconomic drivers of bushmeat consumption

The socioeconomic literature on bushmeat is dominated by poverty, arguing that poorer households rely on wild foods due to their lower cost, more accessible, and less capital-intensive nature (38, 47–49). This interpretation is well supported, but only reflects one aspect of the broader bushmeat economy. Urban bushmeat markets serve wealthier consumers who appreciate wild game as a delicacy or a status symbol, and are willing to pay higher prices for it (35, 50–52). These two distinct types of demand systems are connected to the same trade network, but react to different economic incentives.

The impact of this dual structure is that interventions to address supply-side poverty might be less successful without larger structural shifts in the demand for products and the economic opportunities. If rural livelihood improvements reduce the number of hunters, wealthier urban demand will sustain prices at a level that keeps the trade viable. Market chain assessments also reveal that all the actors in the chain (hunters, middlemen, and market retailers) obtain major income from the trade, thus increasing distributed economic dependency that can lead to resistance to sudden and radical regulatory changes (53–55). Ordaz-Nemeth *et al*. (38) noted the critical point that even when Ebola was an active outbreak, lower-income households who had limited choices of protein alternatives did not significantly reduce consumption despite the awareness of the health risk. This discovery indicates that, in the absence of affordable and culturally appropriate alternatives, raising risk awareness may not be enough to change behavior. Under these circumstances, exposure to zoonotic risks can continue not because people are uninformed, but due to structural barriers that limit their ability to modify consumption practices. The policy implication is that, to achieve sustainable reduction in bushmeat consumption, there is a need for good economic alternatives that are accessible and affordable, such as cheap livestock alternatives, diversified livelihood opportunities, and wider investments in the food system that tackle the structural conditions which underpin the bushmeat dependency (56). Therefore, understanding the socioeconomic diversity of bushmeat markets is key to the design of contextually relevant interventions that can help mitigate zoonotic spillover risk.

## 5 Zootherapeutic practices and their cultural significance

Zootherapy has received less focus than bushmeat consumption in the public health literature, despite being an important pathway for zoonotic pathogen exposure. However, the use of animal parts for medicine, spiritual, and cultural purposes is common in African communities, and creates exposure pathways that are distinct from food consumption (57–59). In Southern Ethiopia, reptile fats and bird parts are used for treating skin conditions and rheumatism (60). Some organs, bones, and skins of primates are used in healing rituals and protective charms in parts of Cameroon (61, 62). Likewise, in Benin and Togo, pangolin scales and snake oil are sold to promote fertility and protection from spiritual problems (63, 64). In particular, the animal species that are currently most demanded by people in these practices are highly similar to the species known to be reservoirs for zoonotic pathogens (65), suggesting that cultural and medicinal uses of these species may inadvertently expose humans to biologically important sources of infection.

Bushmeat activities and zootherapy practices are major avenues for zoonotic disease exposure, even though they may do so via different routes. Bushmeat exposure can happen during the capture and handling of animals via bites, scratches, and contact with saliva, blood, and feces, and also during the slaughter, evisceration, and processing of carcasses and offal. Zootherapy practices can involve the repeated handling and processing of animal materials such as organs and blood and nervous tissue, often also without protective equipment (60, 66, 67). For the reasons mentioned, risks should not be assigned based on one of the exposure routes. Zoonotic pathogens, including *filovirus*, are especially present in the nervous tissue and blood of primates, which makes them a high risk to hunters and healers who process them in ritualistic activities (68). Surveillance data from Ghana also identified risks of exposure via bat-derived materials used in traditional medicine (69).

Zootherapy has been identified recently as a long under-appreciated area for exposure risk in a recent One Health evaluation by Friant *et al*. (58). However, evidence linking specific human infection to zootherapeutic practices remains limited. Most of the studies are ethnobiological surveys, qualitative interviews, and descriptive surveys, and do not rely on serological or virological data; therefore, difficult to determine the frequency of these exposures that lead to human infection. This gap has a practical implication for surveillance and intervention design. Because zootherapeutic practices are private and ritual in nature, they are poorly captured by standard epidemiological surveillance. The demand they generate also sustains hunting pressure on high-risk species of wildlife, which could continue to pose zoonotic spillover risk even if bushmeat markets are restricted or regulated (68). Although regionally, there may be significant links between these pathways and the diversity of zootherapeutic practices in Africa, the extent of this diversity and its connection to wildlife trading networks is not well documented. To fill this gap, a new interdisciplinary research initiative is needed, combining ethnographic research with pathogen surveillance and public health risk assessment to better understand the extent and consequences of zootherapeutic exposure.

## 6 Changing cultural norms and their implications for zoonotic risk

The cultural norms of eating bushmeat and using zootherapy practices are not fixed but changeable; however, the magnitude and nature of these changes differ greatly among regions, and existing literature provides a complex and sometimes contradictory picture. For instance, in Nigeria, bushmeat, especially grasscutter and antelope, continues to have high cultural value as a marker of taste, tradition, and social identity (70, 71). In the same country, evidence suggests that urban youth increasingly associate bushmeat with illegality or health-related risk and prefer farmed meat as an alternative (37, 52). These findings are not necessarily contradictory; it is probably due to a generational, educational, and geographical difference in social norms. But the issue is that these subgroup differences are not fully captured in the aggregate studies, and therefore interventions developed for a generalized population may not be effective if they fail to consider social or demographic differences.

Religious change has influenced the consumption of wildlife patterns in some settings. Normatively, Islamic teachings have prohibited the consumption of some animals, such as non-human primates and bats, due to their ritual impurity or potential harm (72). Bushmeat trading data in Guinea, West Africa, indicate that at least in one Muslim-dominant African setting, the volume of trade in these species has changed over time, in a manner that may have been shaped by such norms, but without causal evidence (73). There are also reports of the consumption and trade of these species in some West and Central African countries, raising questions about whether comparable religious dynamics may influence consumption practices in African Muslim-majority communities. Furthermore, it is unclear whether such changes would produce sustained reductions in zoonotic risk.

The 2013 to 2016 Ebola outbreak in Guinea, Liberia, and Sierra Leone provides an important case study for examining how cultural norm change is addressed in public health emergencies. In the outbreak period, several governments adopted blanket prohibitions on eating and trading bushmeat, which directly conflicted with traditional dietary habits and livelihoods. These restrictions were found to have influenced the growth of clandestine trade, reduced the trust in health facilities, and compliance by the affected communities (74). While such enforcement measures may have reduced opportunities for effective surveillance and early detection. Conversely, interventions that are co-designed with traditional leaders and that offer viable protein alternatives are more acceptable to communities and sustainable in the long-term (75, 76). Wholistically, literature shows that participation and culturally sensitive methods of changing cultural norms may be more successful in reducing the risk of zoonotic spillover than enforcement-based methods. However, the continued preference for rapid and enforceable methods in many organizations raises the question of whether the problem lies with a lack of evidence or rather the persistence of structural and political incentives that favour visible forms of interventions over slower community-engaged approaches. Understanding how cultural norms evolve and the ways in which interventions influence evolving cultural norms becomes crucial in devising effective measures to reduce zoonotic risk.

## 7 Climate variability, ecosystem disruption, and zoonotic spillover risk

### 7.1 Ecosystem changes and disease ecology

The impact of climate variability on the African ecosystems is well documented. Temperature changes, persistent droughts, floods, and unpredictable rainfall patterns have brought about changes in vegetation, hydrological patterns, and species distribution, affecting pathogens and host dynamics (77–82). According to Suu-Ire *et al*. (83), these changes influence the geographical distribution, survival, and reproductive success of both pathogens and hosts, resulting in increased chances of transmission in areas where it has been minimal before. Linkages between zoonotic spillover events documented in African contexts and specific climate variables, however, are largely indirect and inferred. Most of the studies establish biological plausibility and ecological association rather than direct causal pathways, and the simultaneous occurrence of agricultural expansion, deforestation, and climate change makes it methodologically difficult to isolate which factor is doing the most work in any given setting (84).

These complexities have implications for the design of the surveillance. A climate monitoring system that does not include land-use and socio-economic data is likely to be less effective in its ability to accurately identify or interpret emerging zoonotic spillover risks. Climate-disease relationships are true, but does not operate independently of the human system that surrounds them. It should be noted that the climatic conditions outlined in this section are known, but the pathways downstream that will be compromised by the disruption of ecosystems and lead to higher exposure to zoonotic pathogens are not adequately described. While shifts in climate conditions, including warming temperatures and changes in precipitation, have been well documented, the conclusions about zoonotic spillover risk drawn in this review are based on mechanistic plausibility and cross-contextual consistency across different settings. This is important for understanding current results and for designing the proper targeted surveillance strategies.

### 7.2 Wildlife range shifts and human contact

As habitats become less suitable, wildlife shifts its range, often toward human settlements and agricultural land (85–87). Habitat fragmentation accelerates this by compressing animals into smaller, increasingly human-adjacent areas (88). These ecological changes can lead to more contact between humans and wildlife and novel interfaces for zoonotic pathogen transmission. In West Africa, environmental degradation caused by climate change, along with agriculture, has rendered rodents and small mammals vulnerable to changes in their habitats and brought them to cultivated fields and areas around human settlements. This increases the likelihood for bushmeat hunting and the frequency of incidental human contact during normal farming activities, a pattern documented in Nigeria and Ghana with measurable associations with exposure to rodent-borne pathogens (12, 69, 89). Seasonal livestock migration also brings increased interaction between humans, livestock, and displaced wildlife, resulting in new interaction zones between humans, livestock, and wildlife (90, 91). Changes in grazing movements, as pastoralists respond to environmental pressures, can lead to novel multi-host interactions which could allow pathogen exchange between previously distinct ecological systems. Ecological mobility and human livelihood adaptation must be incorporated into zoonotic risk assessments as highlighted by these developments.

### 7.3 Climate-induced migration as a zoonotic spillover pathway

There are two major ways in which climate-induced human migration can contribute to zoonotic risk. First, it moves people who lack established wildlife exposure norms into places with frequent and normalised contact with reservoir hosts (9, 90), for instance, peri-urban communities close to existing bushmeat markets and forest-margin communities where hunting is common. Secondly, it establishes new pathogen bridges across ecological areas, as migrants moving between regions carry their exposure history and possible infection across previously separate ecological systems. This hypothesis is supported by converging evidence from disease ecology, migration studies, and African field research, even though it has not yet been tested in longitudinal studies.

Given the policy implications that follow from this hypothesis, it is useful to consider three tiers of evidence. Tier one comprises findings replicated across multiple datasets: climate variability displaces African populations, and this is documented robustly across subregions (92–94). Tier two comprises patterns that are biologically plausible and consistent with emerging field data but not yet causally established: displaced populations entering peri-urban bushmeat trade networks face elevated zoonotic exposure risk. This proposition is supported by the structural logic of trade networks and migration corridors, and by emerging field observations (92), but a dedicated longitudinal study linking displacement events to subsequent infection outcomes has not yet been conducted. Tier three is propositions whose main argument is based on inferences from the arguments of tiers one and two: climate-induced migration is already having measurable effects on population-level rises in zoonotic spillover probabilities. This is plausible given the preceding evidence, but not yet proven in practice. It is important to distinguish these tiers to help clarify which conclusions are actionable now and which need additional evidence before they can responsibly anchor policy.

Changes in land use can create new places for humans and wildlife to interact. Mining, logging, farming, and creating plantations and large monocultures can displace workers to habitats less disturbed by humans. Associated migration can cause contact with wildlife and greater movement of people. These activities can also disrupt wildlife habitats and change their behavior, consequently increasing contact with humans. These activities can also disturb wildlife. This can create the conditions for the spillover of zoonotic pathogens, especially forest edge environments (95–97).

The limited evidence available suggests a consistent impact from climate-driven human mobility on the geographic distribution of disease exposure risk, beyond what can be detected through static surveillance, as shown by Glidden *et al*. (93). Specific instances of communities displaced by climate change becoming part of peri-urban bushmeat trade networks in West Africa are reported in Edward *et al*. (92), and there are detectable shifts in exposure profiles. The findings cannot yet establish causation at a population level, but are mechanistically plausible and consistent across different research contexts. The limitation in the available evidence resides not in the internal inconsistency but in insufficient density to support definitive conclusions.

The practical question is what should be done in the gap in the evidence. What follows is our recommendations rather than a further empirical finding; we judge the mechanistic plausibility of the migration-spillover pathway is now strong enough to justify designing surveillance systems that can capture it, rather than waiting for retrospective evidence from a future outbreak. Specifically, we propose that climate early warning systems should be connected to monitoring systems in the trade corridors in the receiving areas; serological screening programs should look for recent arrivals to peri-urban settlements near active bushmeat markets; and migration corridor data from the Sahel, the Lake Chad basin, and the Great Rift Valley should be formally included in national One Health risk assessments. The data systems needed already exist, but the most important challenge might be to enhance the cross-sector integration of data systems and institutional coordination.

## 8 Driver interactions and compound zoonotic spillover risk

Existing literature on zoonotic spillover in Africa mostly addresses cultural practices, wildlife trade, and climate change as distinct avenues of research. This separation is convenient in analysis, but epistemologically problematic in practice: the significant zoonotic spillover risks arise at the intersections and across the interplay of these drivers.

The Sahel and sub-Saharan West Africa are a good example. Repeatedly occurring droughts lead to lower agricultural productivity and livestock survival, thus increasing the dependence of households on bushmeat hunting as a coping mechanism. This increased hunting pressure targets the killing of small mammals and rodents, which are also the main reservoir host for the Lassa virus. At the same time, climate change is expanding the geographic range and population densities of these reservoir species (83, 89). This results in the convergence of greater human hunting activity with a larger climatically induced reservoir population. Neither a purely cultural analysis nor a climate analysis would reveal this dynamic; it only becomes visible when the economic stress driving hunting decisions is read alongside the ecological changes shaping reservoir distribution.

The second important intersection is in the context of trade networks and institutional responses. Public health crises bring enforcement to bear on bushmeat markets, which results in the displacement of trade. This decreases the visibility of the market at precisely the moment when pathogen monitoring is crucial (74). This may create a feedback loop in which efforts intended to reduce high-risk trade inadvertently degrade the surveillance capacity needed to detect and contain the very zoonotic spillover events. This has been observed during the Ebola response in West Africa, and is a structural feature of informally organized trade systems that recurs regardless of the pathogen involved (53, 74).

The third intersection works on migration and trade network entry. When climate-displaced individuals settle in peri-urban areas close to functional bushmeat markets, they enter high-exposure environments where there are no occupational norms or community risk practices. This aligns with emerging disease ecology frameworks (92, 93). Understanding this pathway requires research designs that track climate migration and trade participation, and infection outcomes.

## 9 Wildlife trade: markets, pathogens, and governance

### 9.1 Market structure and epidemiological implications

**Figure 4:**
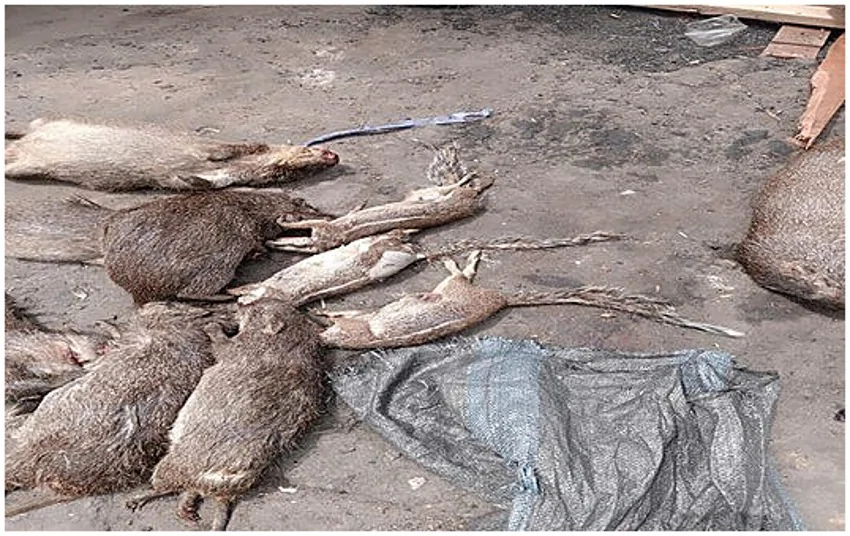
Rodents displayed for sale at a bushmeat market in Kumasi.

**Figure 5:**
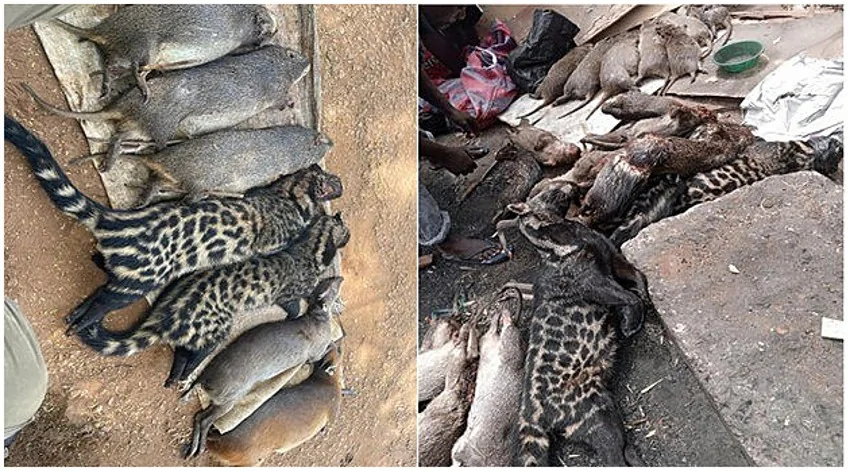
Rodents and wild cats displayed for sale at a bushmeat market in Kumasi.

**Figure 6:**
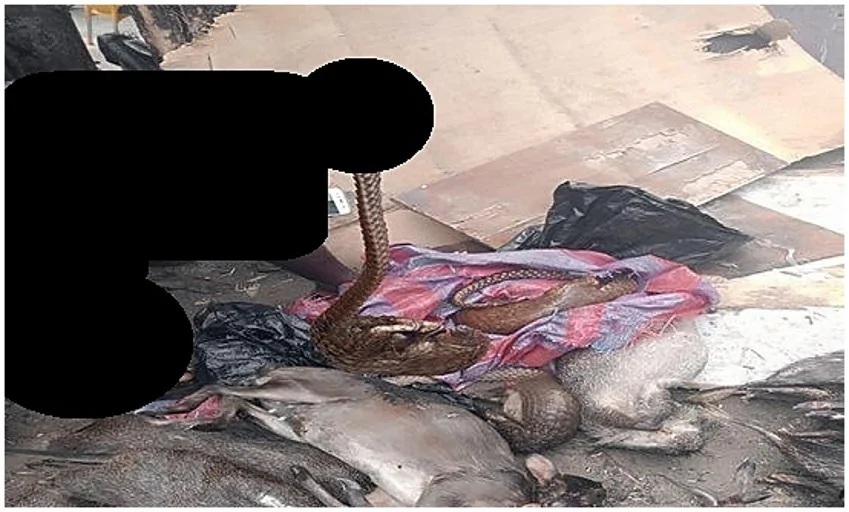
Pangolins and rodents displayed for sale at a Bushmeat market in Kumasi.

Bushmeat markets range from small rural stalls, where freshly hunted animals are sold directly to local consumers (36, 98), to large urban consolidation hubs in cities such as Douala, Bertoua, Kinshasa, and Bauchi, Lagos, where game from multiple ecological zones is aggregated for higher-value sale (35, 99). This spatial structure has direct epidemiological consequences: when wildlife from different hunting zones converges at a single urban market, a pathogen present in one region can reach multiple consumption areas simultaneously. The character of these markets, including the diversity of species handled, the physical conditions of sale, and the volume of animal material traded, is illustrated by field photographs from a Kumasi bushmeat market (Figures 4–6), which document the simultaneous sale of rodents, wild cats, and pangolins, all known reservoir taxa for zoonotic pathogens. Understanding the structure and movement patterns of this trade system is therefore not merely a matter of trade policy or governance; it is also a foundation for the development of effective disease surveillance and early warning systems.

The gendered structure of these markets has been noted in the literature but not adequately investigated, and the fragmented evidence that does exist warrants more careful synthesis than it typically receives. Women dominate retail trade in urban markets while men predominate in rural hunting supply (46). This division of labour matters for exposure risk because the activities are not equivalent: hunters face acute, episodic exposure during slaughter and initial processing, while urban retailers face sustained, daily exposure through repeated handling of carcasses, blood, and bodily fluids across multiple species and ecological origins. McNamara *et al*. (54) and Bachmann (53) document that women traders often handle higher volumes of animal material per day than hunters do on a typical hunting trip, yet their role in the trade chain is systematically less visible to health surveillance. The few studies that have occupational exposure data stratified by sex, including those from Ghana’s bushmeat markets (69) and Central African trade networks (39), consistently identify female market traders as a high-contact population; however, none have proceeded from this observation to serological or virological examination of exposure outcomes. If women who handle and sell bushmeat face disproportionate occupational zoonotic exposure, which the structural logic of their role strongly suggests, then gender-blind surveillance will systematically undercount infection risk in this population. This is not solely an equity consideration. It represents a systematic surveillance gap in outbreak detection that has been identified in the literature for over a decade, without generating a single dedicated serological study in which female market traders constitute the primary study population.

### 9.2 Pathogens in the trade: what the evidence shows and what it cannot tell us

Pathogen surveillance in traded African wildlife has produced valuable findings, but the three categories of pathogens documented carry very different risk profiles that the literature rarely examines side by side. Viral pathogens detected in non-human primates, including primate T-lymphotropic viruses (PTLVs), poxviruses, and retroviruses, require close and direct contact with blood or tissue to infect humans (25, 41, 68, 101). These exposures represent lower-frequency but potentially high-consequence transmission events concentrated among hunters and butchers. In contrast, rodent-associated viruses such as Lassa and novel rhabdoviruses identified in West African surveillance represent a different risk profile: broader population exposure, including among farmers and communities living in proximity to wildlife, linked to both the trade and everyday environmental contact (100–103). A third profile of bacterial pathogens recovered from wildlife sold in markets is pathogens that are more frequently transmitted, but with a lower individual risk of infection, such as *Campylobacter, Salmonella,* and *Shigella*, which are poorly discussed in public health literature on zoonotic spillover despite being more commonly transmitted (40, 104). Although the public health implications of these three profiles are very different, most surveillance programs and risk communication frameworks treat them under a single concept of bushmeat risk.

There are two important structural weaknesses in the surveillance literature that deserve special attention. First, bats are known reservoir hosts of many high-consequence zoonotic viruses such as *filoviruses* and *coronaviruses*, but there are limited market surveillance data of bats compared to the known risk (65, 69). Partly because trade in bat species is carried out in less visible ways than larger mammals, such as in zootherapeutic markets. The lack of surveillance data does not reflect an absence of risk. Secondly, the entire evidence base is geographically clustered in countries with functioning research infrastructure, such as Ghana, Nigeria, Uganda, Kenya, and the Democratic Republic of Congo. The literature on surveillance is limited in fragile and conflict-affected settings, where the informal wildlife trade may be more active. Given that outbreak risk is likely higher in precisely these settings, the evidence base is systematically skewed away from contexts that matter most. Most importantly, across all settings, surveillance studies report the presence of pathogens and not the probability of their transmission. We know what pathogens are circulating in traded wildlife, but we do not know what handling conditions, interactions in the market, or consumer practices convert exposure into infection. Risk communication and intervention strategies will continue to rely on an incomplete epidemiological basis until there is a better understanding of the conversion of exposure to transmission.

### 9.3 Domestication as an alternative to bushmeat consumption: a critical assessment

Domestication of selected wild animal species has increasingly been promoted as a strategy to reduce reliance on bushmeat and enhance food security and livelihoods. Grasscutter domestication has reportedly been a considerable success in West Africa in terms of farming viability, consumer acceptance, and market integration in Ghana (105, 106), Nigeria (107, 108), and Benin (109). Similarly, the possibility of sustaining a livelihood through rabbit production in peri-urban communities has also been noted, as it generates income and dietary income security (110–112). These interventions offer socio-economic opportunities and deserve to be maintained in a wider context of food security and livelihood development. However, the case for domestication as a zoonotic spillover prevention strategy, which is often presented in the literature, relies on an assumption that has not been tested. This argument assumes that farmed wildlife will replace the hunted bushmeat and that this will significantly lower exposure to zoonotic pathogens. However, the empirical basis for this assumption remains limited.

The conditions under which domestication would offer significant risk reduction, specifically biosecure smallholder operations with limited contact between farmed animals and wild reservoir populations, are not the norm in practice. In West Africa, peri-urban smallholder farms share ecological space with wild rodent populations, rely on shared water resources, and often have poor access to veterinary services. Under such conditions, farmed grasscutters may not be free of the pathogen exposure routes associated with their wild counterparts. This is not a hypothetical problem; it is a concern that is documented in smallholder livestock farming in West Africa (112). Other barriers further limit the ability of domestication programs to be considered successful alternatives to bushmeat. High upfront costs, limited access to credit, poor cold chain facilities, and market volatility further limit the adoption and long-term sustainability (110). Meanwhile, consumer demand and cultural preferences for the taste and prestige associated with wild meat persist even where affordable options are available (56, 113). These factors suggest that domestication may supplement existing food systems without necessarily displacing demand for wild-sourced bushmeat.

Most fundamentally, domestication does not address zootherapeutic demand for wild animal parts, including species and biological materials that cannot be substituted by any conventional farming. Hunting pressure on the high-risk taxa valued in traditional medicine continues regardless of how well food domestication programs perform. Viewed together, the available evidence suggests that domestication is a valuable intervention for improving livelihoods, strengthening food security, and potentially reducing some forms of dependence on bushmeat. However, its effectiveness as a direct zoonotic spillover prevention strategy remains unproven. It should not, however, be presented or funded as a zoonotic spillover prevention strategy until evidence from field conditions, rather than controlled pilot programs, demonstrates that it actually reduces human exposure to zoonotic pathogens. Positioning domestication as a prevention strategy in the absence of such evidence risks reducing its preventive efficacy to a partial solution and diverting attention from interventions that address zoonotic spillover risk earlier in the wildlife-human transmission chain.

## 10 Efforts to reduce zoonotic spillover: progress and persistent failures

### 10.1 What has been achieved

Repeated outbreaks of Ebola, Lassa fever, Rift Valley fever, anthrax, rabies, and COVID-19 have pushed zoonotic spillover onto Africa’s institutional agenda in meaningful ways (23, 114, 115). Uganda, Nigeria, Kenya, and Ghana, supported by WHO, FAO, and Africa CDC, have conducted national zoonotic disease prioritisation exercises and built integrated human-animal surveillance platforms (116, 117). Regional bodies such as ECOWAS and the East African Community have facilitated cross-border surveillance and information sharing, acknowledging that wildlife movements, livestock trade, and human migration are inherently transboundary (118). One Health platforms are now implemented with FAO, WOAH, UNEP, and Africa CDC support across multiple countries (119).

### 10.2 Where one health needs to be strengthened

The areas discussed immediately below, together with the structural barriers discussed subsequently, combine three levels of inference that we distinguish explicitly. The institutional record on the 2013–2016 bushmeat ban and the documented under-resourcing of wildlife surveillance are directly evidenced in the cited literature. By contrast, the characterisation of community knowledge systems as a comparatively neglected third pillar is our interpretation of a pattern across these sources, since no single study measures this neglect directly. The implications we draw for how One Health platforms should be redesigned are, similarly, our own recommendations, informed by but not established by the evidence, and are flagged as such where they appear.

While significant institutional progress has been made in the implementation of One Health in African contexts, persistent structural challenges remain, which limit its effectiveness in preventing zoonotic spillovers. One clear limitation was evident during the 2013–2016 West African Ebola outbreak, when several governments used blanket bans on bushmeat as risk reduction strategies. These measures directly conflicted with local food practices and livelihoods, and led to poor compliance and the displacement of bushmeat trade into informal and less visible channels. These enforcement policies actually weakened surveillance systems, made it easier for pathogens to spread, and undermined trust in health authorities (74). The outcomes reflect the limitations of interventions that do not consider social and economic realities, a challenge that remains relevant in the current outbreak response interventions (120).

A second reported area to strengthen concerns about wildlife surveillance in One Health platforms. The concept of linking human, animal, and environmental health is conceptually incorporated into the framework, while the action on wildlife health has proved to be the weak link. Most African countries lack a formal health system that is connected to the wildlife surveillance effort, which is often underfunded and understaffed (118, 119). As a practical matter, zoonotic spillover events are only identified after human-to-human spread occurs, when containment costs are significantly greater, and the opportunity to prevent the spread earlier in the transmission chain has been lost.

A third, more basic, and under-discussed area is that of the neglect of community knowledge systems over biomedical surveillance and laboratory detection in the implementation of One Health in Africa (120). The most direct actors in zoonotic risk, such as hunters, market traders, and traditional healers, whose observations could significantly improve early detection, are often positioned as targets of intervention rather than co-producers of the knowledge that interventions require. This is not simply a design oversight. It reflects a structural imbalance in the valuing of knowledge in global health systems and has substantive operational implications, including poor quality of surveillance data, poor acceptability of interventions, and further fuelling of community mistrust of mistrusted institutions.

### 10.3 Structural barriers that go deeper than funding

Funding gaps and weak governance are cited as the major challenges in the global perspective for strengthening One Health in Africa. While these constraints are substantive, they are not necessarily the most basic structural constraints, and addressing them in isolation without considering the root causes can create solutions that leave the underlying institutional structure intact. In certain African jurisdictions, wildlife trade networks involve actors and groups with substantial economic and political power, such as commercial traders, traditional medicine providers, and cross-border trade networks, whose activities yield financial gains that may be partially captured by the enforcement actors that are tasked with regulating them (53, 54). Weak enforcement in these environments may be due to a stable political arrangement rather than a simple capacity deficit. If programs are designed without consideration of this political economy, they are likely to be superficially complied with, and this will result in a masked continued practice.

The second structural barrier concerns the nature and origin of knowledge. One Health messages built around viral evolution analysis, ecological modelling, and laboratory-confirmed transmission chains are remote from the experiential frameworks of communities whose understanding of disease causation is relational, spiritual, or socially embedded (121, 122). This imbalance is not amenable to resolution through improved visual communication materials alone. It represents an authentic, different way of understanding health and risk, which requires collaborative, community-driven knowledge development rather than translated information delivery.

The third structure is the deliberate concealment that organizes informal bushmeat and zootherapeutic markets. These activities are designed to be undetectable, meaning that standard active and passive surveillance will consistently undercount exposures. In our assessment, addressing this concealment would require sentinel surveillance systems that actively involve trusted members of the community, such as healers, market leaders, and hunters’ associations, as active surveillance partners and not merely notification sources.

## 11 The integrated one health approach

One Health has emerged as the key framework for the prevention of zoonotic spillover, developed from the understanding that the three domains of human, animal, and environmental health are interdependent (123, 124). In Africa, this has been evidenced by an aspiration to shift from outbreak response to upstream prevention measures and interventions related to ecological disruption, wildlife trade, climate variability, and socio-cultural practices, with the support of multiple international partners (115, 119), through joint surveillance systems, integrated laboratory networks, and cross-sectoral training. Several countries have established national platforms that coordinate and share data and conduct joint assessments of risk between the ministries responsible for health, agriculture, wildlife, and environment (118).

The framework has evolved in a positive way, including the integration of behavioral and social sciences, which recognizes that changing cultural practices requires more than biomedical messaging (120). Another truly novel development is the incorporation of ecological and predictive modelling to help predict shifts in wildlife distribution and new potential zoonotic spillover hotspots (125, 126). However, as the critical analysis shown above elaborates, many of these advances remain aspirational in many African contexts. The gap between One Health as a conceptual framework and One Health as a functioning operational system is wide. Closing this gap, in our view, would require addressing the political economies that enable high-risk wildlife trade, integration of community knowledge systems into surveillance architectures, and linking economic opportunities to risk reduction, something that current wildlife trade funding programs often fail to do.

## 12 Limitations

This review is not a systematic or scoping review; selection bias cannot be entirely ruled out. Available evidence was not evenly distributed. Many studies came from Ghana, Nigeria, Cameroon, the Democratic Republic of Congo, and Guinea, while fragile and conflicted regions, where informal wildlife trade and human–wildlife contact may be substantial, remain comparatively underrepresented in the published literature. This geographical limitation reduces the generalizability of the results and might underestimate the zoonotic spillover risk in less explored areas. Future reviews should be explicit in their inclusion of alternative evidence streams such as key informant networks, remote sensing data on deforestation and human movement, cross-border NGO health records, and oral disease history methodologies, rather than treating their absence from peer-reviewed literature as an absence of risk. Due to the fact that many practices listed are clandestine in nature or culturally sensitive, a substantial amount of the review is based on qualitative studies, case reports, and grey literature, which may not fully reflect the extent or dynamics of these practices. Finally, the COVID-19 era has likely changed many of the dynamics outlined above, in ways that are not yet reflected in the existing literature. COVID-19 disrupted bushmeat market operations and supply chains in multiple African countries, in some cases temporarily reducing trade volumes and in others displacing trade to less monitored channels. The pandemic also affected institutional trust in health authorities and community willingness to adhere to the changes in the surveillance program that are the basis for the arguments made in this review for intervention feasibility.

## 13 Conclusion

Zoonotic spillover in Africa does not happen spontaneously. It is a structured product at the intersection between culturally embedded practices of the wildlife and economically organized trade networks and ecologically disruptive climate change. Each driver reinforces the others via processes that are missing from a systematic analysis by a single driver. Climate-related livelihood stress can lead to hunting reservoir species, the concentration of hunting networks generates more regional exposure to the risks leading to pathogen transmission, and institutional interventions to address these risks inadvertently push high-risk practices into clandestine channels. Effective management of zoonotic spillover risk is not achieved by tackling the individual drivers alone, but rather by working at these intersections.

## 14 Recommendation

The following recommendations are grounded in the evidence reviewed and differentiated by subregional context. They should be read as our informed interpretation of what the reviewed evidence implies for practice, rather than as established conclusions or as interventions that have themselves been tested; the feasibility notes given below reflect the same judgement-based status.

Priority 1–Redesign surveillance to include the most under-monitored exposure pathways: Across all subregions, the most significant gap in surveillance is the near absence of monitoring of zootherapeutic handling chains. Traditional healers, hunters who provide medicinal parts, and market traders who handle more repeatedly than most bushmeat consumers, yet they appear in no formal surveillance structure. The most important priority this review identifies is the integration of these actors as paying sentinel surveillance partners instead of unpaid informants. In Central Africa in particular, this would offer an earlier warning of the zoonotic spillover of *filoviruses* than any current system can offer. On feasibility: there are compensation mechanisms in place for informal sentinel partners in precedent community health worker programs in other parts of the continent; the administrative challenge is not new. The more salient constraint is data governance: communities and healers will not be likely to be truly engaged until they have negotiated and established clear protocols for the storage, sharing, and use of surveillance data. The establishment of these governance structures should be mandatory for platforms included in National One Health structures as a prerequisite for international funding, and not seen as potential improvements.

Priority 2–In southern Africa, informal cross-border trade zones: The first and crucial action in this zone is not an intervention but rather the careful mapping of the structure of the trade networks. No existing trade network analysis is available that documents who is trading what wildlife products, through what routes, across what borders, and who is moving what wildlife products. Historical approaches of enforcement only in Zimbabwe and South Africa, as well as in Mozambique and Zambia, have caused traders to move to less monitored corridors. This has not led to a reduction in risk but has produced geographic diffusion. To prevent such results in any future enforcement action, it should be accompanied by alternative livelihood programs for displaced traders, rather than implemented after enforcement. The health service with explicitly health service-oriented serological screening, co-designed with border communities, provides a more acceptable point of entry rather than a surveillance operation. Regarding feasibility: this type of trade network mapping is well within the methodological repertoire of regional research institutions in Southern Africa, and several of which have conducted such analyses for conservation purposes.

Priority 3–Climate migration monitoring should be actioned into all platforms: All national One Health platforms should formally integrate climate migration monitoring into risk assessment processes. This involves connecting existing climate displacement monitoring information that some African governments are already gathering to trade network monitoring and serological surveillance in peri-urban receiving countries. It requires health, climate, and migration and transit authorities to be open to sharing data in ways that they are not currently structured to do. This can be achieved through coordination, which is more achievable than voluntary interagency coordination, and can be leveraged by international funders as a condition to support the platform. On feasibility: the Internal Displacement Monitoring Centr (IDMC) and IOM’s Displacement Tracking Matrix already gather relevant/needed mobility data in several countries in Africa where IOM operates; the challenge is integration, not data gathering. An entry point for implementation would be a joint working group under the One Health coordination structures of Africa CDC to develop a standard protocol for linkage between displacement triggers and surveillance activation, which would then be implemented by the country platforms without each having to develop its own protocol.

## 15 Implementation challenges and feasibility considerations

One Health interventions could prove mutually beneficial. However, in Africa, the comprehensive integration of One Health systems would likely face logistical, economic, and political hurdles. Integrated systems would require significant investments, which developing nations may not be able to afford. This would target the development and enhancement of laboratory, Health, and data systems infrastructure and personnel, and transport systems. Additional integrated health, veterinary, environmental, and Agricultural systems would face challenges of a fragmented workforce, disintegrated and competing policy Health systems, and a lack of data. Integrated community approaches would be hindered by a subsistence economy (derived from the consumption of wildlife and untapped resources), impoverished socioeconomic conditions, traditional practices, poor relations and confidence in public authorities, and the absence of alternative means of subsistence. Although there may be restrictions on wildlife use and trade, enforcement of such constraints may be ineffective in areas where a subsistence economy exists and provides an informal means of trade. There may be climate-related behavioral constraints, such as migration, which would also prove problematic, as adequate interventions would require resource dependence and participation across nations with divergent approaches and goals. Requisite policies then need to be carefully adapted to the prevailing systems and be resource-dependent, while considering their impact on public health.
